# Acute confusional state in paediatric age - Case Report

**DOI:** 10.1192/j.eurpsy.2023.1509

**Published:** 2023-07-19

**Authors:** F. Cordeiro, T. Cartaxo, F. Reis, P. Moniz, M. Alves, C. Bayam, V. Santos

**Affiliations:** Serviço de Pedopsiquiatria, Hospital Pediátrico CHUC, Coimbra, Portugal

## Abstract

**Introduction:**

Acute confusional state (ACS) or delirium is an acute neuropsychiatric syndrome due to an underlying organic pathological process. Despite its high prevalence, delirium can present a diagnostic challenge, particularly in paediatric patients. ACS can be defined as sudden impairment of mental status in a previously healthy child. The impairment varies; it may be global and severe or very specific and mild, such as impairment of short-term memory in “transient global amnesia.” The most common causes of ACS in the paediatric population are high fever, drugs, traumatic brain injury (TBI), and infection and inflammation of the nervous system. Traumatic brain injury is usually associated with some impairment of consciousness, although recovery can vary depending on the severity of the trauma.

**Objectives:**

The aim of this work is to revisit the diagnostic approach and management of ACS associated with traumatic brain injury in the paediatric population.

**Methods:**

Case report of an acute confusional state, secondary to a TBI and a non-systematic review of the literature.

**Results:**

A 17-year-old female was admitted to the emergency department after being injured in a car accident. She was drowsy but easily awakened. She was conscious and partially oriented in time and space. She had amnesia for the episode. She spoke fluently and coherently but was hesitant regarding the hours before the accident, which was probably due to memory impairment. She exhibited sporadic hetero-aggressive behavior during the first few hours of the examination. She had no other thought or perceptual disorders. Head CT scan showed “a thin collection of blood from the frontal interhemispheric area and a discrete subarachnoid sulcal frontobasal hemorrhage, with no other significant changes.” Toxicology tests were positive for THC, cocaine, and MDMD and negative for blood alcohol. A forensic medical examination was required. After 48 hours of vigilance and improvement, she was discharged with a booked re-evaluation within a week. At the second evaluation, her mother described a change in her usual behavior with disorientation, drowsiness, difficulty managing daily life, and memory impairment. She had persecutory delusions regarding the physicians and was very agitated. She was admitted to a child and adolescent psychiatric hospital for further evaluation and stabilization. After 72 hours of inpatient stay, she fully recovered, receiving low-dose risperidone daily. She was discharged with the diagnosis of delirium due to another medical condition (TBI), acute, hyperactive. Since discharge, symptoms have not recurred even after discontinuation of antipsychotic medication.

**Conclusions:**

Clinically, ACS can be divided into hypoactive, hyperactive, and mixed level of activity. Hyperactive forms may manifest as varying degrees of psychomotor agitation. With this case report, we’d like to raise awareness of ACS so that it’s diagnosed and treated correctly and in a timely manner.

**Disclosure of Interest:**

None Declared

